# Natural *Wolbachia* infection in field-collected *Anopheles* and other mosquito species from Malaysia

**DOI:** 10.1186/s13071-020-04277-x

**Published:** 2020-08-12

**Authors:** Meng Li Wong, Jonathan Wee Kent Liew, Wai Kit Wong, Sandthya Pramasivan, Norzihan Mohamed Hassan, Wan Yusoff Wan Sulaiman, Nantha Kumar Jeyaprakasam, Cherng Shii Leong, Van Lun Low, Indra Vythilingam

**Affiliations:** 1 Department of Parasitology, Faculty of Medicine, University of Malaya, 50603 Kuala Lumpur, Malaysia; 2 Department of Biomedical Science, Faculty of Medicine, University of Malaya, 50603 Kuala Lumpur, Malaysia; 3 Entomology & Pest Unit, Kluang District Health Office, 86000 Kluang, Johor Malaysia; 4 Tropical Infectious Diseases Research and Education Centre (TIDREC), University of Malaya, 50603 Kuala Lumpur, Malaysia

**Keywords:** *Wolbachia*, Mosquitoes, *wsp* gene, *16S* rRNA, *Anopheles*, Vectors

## Abstract

**Background:**

The endosymbiont bacterium *Wolbachia* is maternally inherited and naturally infects some filarial nematodes and a diverse range of arthropods, including mosquito vectors responsible for disease transmission in humans. Previously, it has been found infecting most mosquito species but absent in *Anopheles* and *Aedes aegypti*. However, recently these two mosquito species were found to be naturally infected with *Wolbachia*. We report here the extent of *Wolbachia* infections in field-collected mosquitoes from Malaysia based on PCR amplification of the *Wolbachia wsp* and *16S* rRNA genes.

**Methods:**

The prevalence of *Wolbachia* in Culicinae mosquitoes was assessed *via* PCR with *wsp* primers. For some of the mosquitoes, in which the *wsp* primers failed to amplify a product, *Wolbachia* screening was performed using nested PCR targeting the *16S* rRNA gene. *Wolbachia* sequences were aligned using Geneious 9.1.6 software, analyzed with BLAST, and the most similar sequences were downloaded. Phylogenetic analyses were carried out with MEGA 7.0 software. Graphs were drawn with GraphPad Prism 8.0 software.

**Results:**

A total of 217 adult mosquitoes representing 26 mosquito species were screened. Of these, infections with *Wolbachia* were detected in 4 and 15 mosquito species using *wsp* and *16S* rRNA primers, respectively. To our knowledge, this is the first time *Wolbachia* was detected using *16S* rRNA gene amplification, in some *Anopheles* species (some infected with *Plasmodium*), *Culex sinensis*, *Culex vishnui*, *Culex pseudovishnui*, *Mansonia bonneae* and *Mansonia annulifera*. Phylogenetic analysis based on *wsp* revealed *Wolbachia* from most of the mosquitoes belonged to *Wolbachia* Supergroup B. Based on *16S* rRNA phylogenetic analysis, the *Wolbachia* strain from *Anopheles* mosquitoes were more closely related to *Wolbachia* infecting *Anopheles* from Africa than from Myanmar.

**Conclusions:**

*Wolbachia w*as found infecting *Anopheles* and other important disease vectors such as *Mansonia*. Since *Wolbachia* can affect its host by reducing the life span and provide resistance to pathogen infection, several studies have suggested it as a potential innovative tool for vector/vector-borne disease control. Therefore, it is important to carry out further studies on natural *Wolbachia* infection in vector mosquitoes’ populations as well as their long-term effects in new hosts and pathogen suppression. 
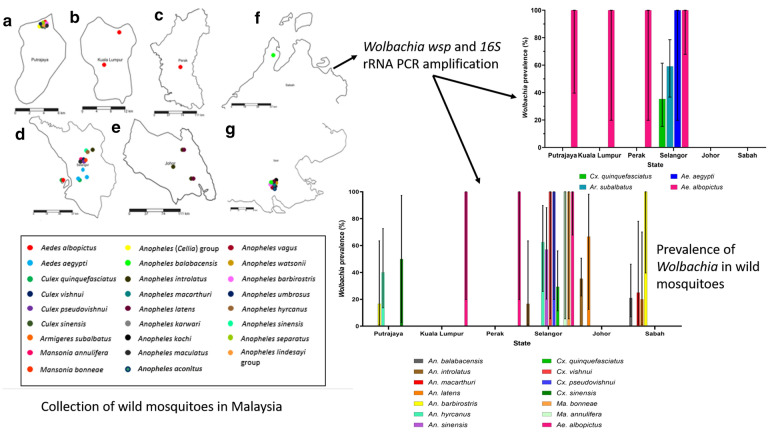

## Background

The endosymbiotic bacterium *Wolbachia* is maternally inherited and naturally infects some filarial nematodes and a diverse range of arthropods, including mosquito vector species which are responsible for disease transmission to humans [1, 2]. *Wolbachia* is found naturally in various mosquitoes, including *Aedes*, *Culex*, *Armigeres*, *Mansonia*, *Coquillettidia*, *Culiseta*, *Hodgesia*, *Ochlerotatus*, *Tripteroides* and *Uranotaenia* [3–8] but was believed to be absent in *Aedes aegypti* and *Anopheles* mosquitoes [3]. To enhance their spread across the population, *Wolbachia* has a wide host range as well as wide tissue distributions [9]. Besides, *Wolbachia* can possibly be transferred horizontally between mosquito populations though the mechanism involved is not fully understood [10].

*Wolbachia* infection in the host causes a broad range of abnormal reproductive phenotypes which include male-killing, parthenogenesis, feminization and cytoplasmic incompatibility (CI) [11, 12]. Cytoplasmic incompatibility caused by *Wolbachia* infection is the most well-known and common phenotypic effect and is considered a potential vector control alternative since crosses between individuals with different *Wolbachia* infection status will result in few or no offspring [13]. This effect confers indirect reproductive advantages to the infected females as infected females can produce viable progenies, but uninfected females when mated with infected males produce fewer viable progenies, if any [14]. Therefore, CI, along with maternal transmission of *Wolbachia*, allows *Wolbachia* to spread rapidly through a population [14, 15]. Several factors affect the CI in the host, namely strain of *Wolbachia*, host species, temperature and rearing density [16–19].

Furthermore, some studies have shown that *Wolbachia* can inhibit pathogen replication within mosquitoes [20, 21] such as dengue virus (DENV) [22–28], chikungunya virus (CHIKV) [25, 29, 30], yellow fever virus (YFV) [30], Zika virus (ZIKV) [31–33], *Plasmodium* parasites [25, 34] and filarial nematodes [35]. In studying the impact of *Wolbachia* infection on *Plasmodium* infection in *Anopheles,* transiently and somatically infected *Anopheles gambiae* with *w*MelPop or *w*AlbB *Wolbachia* strains was found to have significant reductions in *Plasmodium berghei* or *Plasmodium falciparum* oocyst infections [36, 37]. Following the increasing findings of natural *Wolbachia* infections in *Anopheles* [38], it was also found that natural populations of *Wolbachia*-infected *An. coluzzii* females have a lower frequency of *Plasmodium* infections than *Wolbachia*-negative individuals [39]. Gomes et al. [40] further showed that naturally infected field *An. gambiae*, has significantly lower *Plasmodium* sporozoite infections. These findings support the potential use of *Wolbachia* in controlling vector-borne disease transmission. Currently, in Malaysia, studies on the release of *Wolbachia*-infected *Ae. aegypti* are ongoing to curb the spread of dengue [41].

It was believed that *Wolbachia* is absent in *Ae. aegypti* and *Anopheles* mosquitoes [3] until the year 2014, when the *w*Anga *Wolbachia* strain was detected in *An. gambiae* collected from West Africa [38]. This was followed by several other discoveries of *Wolbachia* in other *Anopheles* vectors in Africa [40, 42–45] and Southeast Asia (Myanmar) [46]. Moreover, recent studies have also found *Wolbachia* naturally infecting *Ae. aegypti* in the Philippines [47], Thailand [48], Malaysia [49], India [50], Florida [51], New Mexico [52], Texas [53] and Panama [54].

Amplification of *Wolbachia* genes such as *Wolbachia* surface protein (*wsp*), filamenting temperature-sensitive mutant Z (*ftsZ*) and *16S* rDNA using polymerase chain reaction (PCR) has been used to detect *Wolbachia* in the vector. Previous failure in detecting *Wolbachia* in *Anopheles* mosquitoes may be due to limitations in the detection system used such as non-optimal DNA amplification [38]. This has led to the development of a nested PCR amplification of *16S* rDNA region for *Wolbachia* detection in *Anopheles* mosquitoes [39]. A study by Marcon et al. [55] demonstrated that the combination of *16S* rDNA and *wsp* PCR detection increased the detection efficiency and supergroup differentiation whereas detection with *ftsZ* was less sensitive which can lead to false-negative for *Wolbachia* infection mosquitoes.

Thus, this study aimed to determine natural *Wolbachia* infections in different field-collected mosquitoes from several states in Peninsular Malaysia and East Malaysia (Sabah) using *Wolbachia 16S* rRNA and *wsp* gene amplifications, to determine the extent of natural infection of *Wolbachia* in some of these disease vectors and to understand its role in the dynamics of disease transmission.

## Methods

### Mosquito collection

**Fig. 1:**
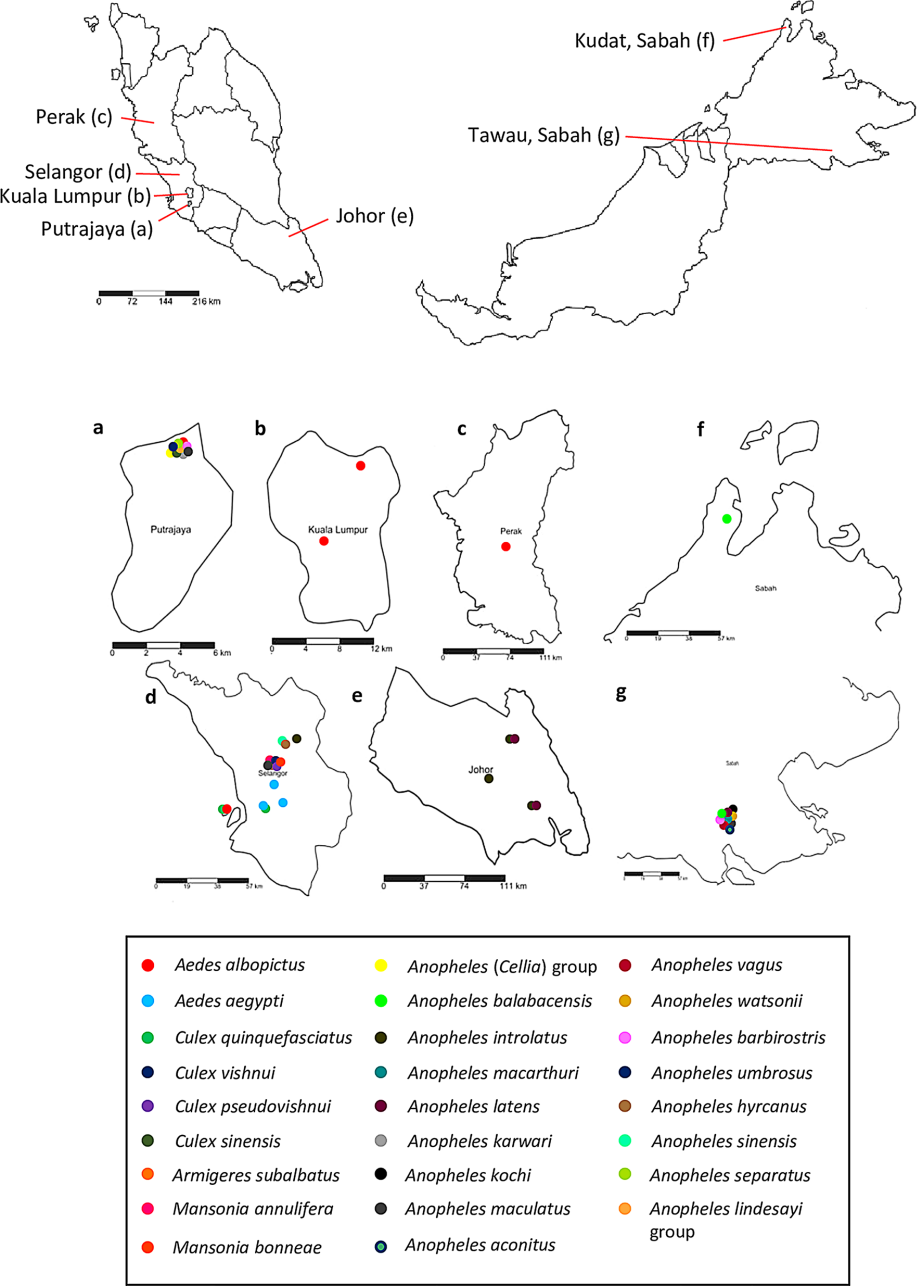
Map showing mosquitoes collected from five different states in Malaysia. **a** Putrajaya. **b** Kuala Lumpur. **c** Perak. **d** Selangor. **e** Johor. **f** Kudat, Sabah. **g** Tawau, Sabah

Adult *Anopheles* spp. and Culicinae mosquitoes collected from 2013 to 2019 in Selangor, Kuala Lumpur, Putrajaya, Johor, Perak and Sabah (Fig. 1) using human landing catch, mosquito magnet trap and sticky trap, were randomly selected and screened for *Wolbachia* (Additional file 1: Table S1). All specimens were identified morphologically using the taxonomic keys of Reid [56], Sallum et al. [57] (Leucosphyrus group of *Anopheles* mosquitoes) and Jeffery et al. [58] prior to molecular detection of *Wolbachia*. Specimens which could not be reliably distinguished based on morphology, such as those of the Leucosphyrus group of *Anopheles* or those which were not well-preserved, were all molecularly identified by PCR and sequencing of the internal transcribed spacer 2 (ITS2) gene [59]. If specimens could not be identified morphologically or molecularly, they were classified to the lowest level of taxonomic certainty, for example the genus level.

Additionally, *Anopheles* samples positive for *Plasmodium* infections were also included in the study (Additional file 1: Table S2). These 34 samples originating from other vector studies had already been screened for simian *Plasmodium* parasites (i.e. *Plasmodium knowlesi*, *Plasmodium cynomolgi*, *Plasmodium inui*, *Plasmodium fieldi* and *Plasmodium coatneyi*) infection by dissection and observation of the midgut for oocysts and salivary glands for sporozoites followed by PCR [60] in well-preserved samples; or by PCR of whole mosquitoes, head/thorax or abdomen in all other samples. The midguts were stained with 1% merchurochrome for visualization of oocysts, while the salivary glands were squashed in saline and viewed under a light compound microscope at 400× magnification for visualization of sporozoites.

### Genomic DNA extraction and molecular detection of *Wolbachia*

Genomic DNA was extracted from the whole mosquito using InstaGene matrix (Bio-Rad, Hercules, California, USA) according to the manufacturer’s protocol. The extracted DNA was run on a 1% agarose gel electrophoresis to confirm its presence. Detection of *Wolbachia* was undertaken targeting two conserved *Wolbachia* genes, *wsp* and *16S* rRNA. *Wolbachia* in Culicinae mosquitoes was screened using general *wsp* primers (*wsp*81F: 5′-TGG TCC AAT AAG TGA TGA AGA AAC-3′; *wsp*691R: 5′-AAA AAT TAA ACG CTA CTC CA-3′), amplified in a 30 µl reaction volume with 5 µl DNA as template according to the standard PCR protocol [61]. These primers amplify a DNA fragment ranging from 590 to 632 bp. Nested PCR amplifying the *16S* rRNA gene was used to detect *Wolbachia* in *Anopheles* and Culicinae mosquitoes whose results were negative in the *wsp* PCR. The initial PCR employed *16S Wolbachia*-specific primers (W-Specf: 5′-CAT ACC TAT TCG AAG GGA TAG-3′; W-Specr: 5′-AGC TTC GAG TGA AAC CAA TTC-3′) and was performed in 30 µl reaction volume using 5 µl DNA as template according to standard protocol in Werren & Windsor [62]. Then, two microliters of the initial PCR products were amplified in a 30-µl PCR reaction using specific internal primers (16SNF: 5′-GAA GGG ATA GGG TCG GTT CG-3′; 16SNR: 5′-CAA TTC CCA TGG CGT GAC G-3′) as described in Shaw et al. [39]. The nested *16S* rRNA *Wolbachia* PCR generates a 412-bp DNA fragment. *Aedes albopictus* (Skuse) was used as positive control for all *Wolbachia* screening along with negative control (double-distilled water as template). All PCR products were run on 1.5% agarose gel electrophoresis and viewed under UV light prior to Sanger-sequencing by a commercial laboratory (MyTACG, Kuala Lumpur, Malaysia).

### Phylogenetic analysis

The nucleotide sequences obtained were aligned using Geneious 9.1.6 software (http://www.geneious.com). All aligned *Wolbachia* sequences were compared with other sequences available in the GenBank database to determine the percentage identity using BLAST (http://blast.ncbi.nlm.nih.gov/Blast.cgi) and the most similar sequences were downloaded for phylogenetic analysis. The alignment of all available sequences was examined visually and only those residues where variations were observed in sequences obtained from independent PCR amplifications from the same sample or from another independently amplified sample were considered reliable and therefore included in the phylogenetic analysis. All sequences (*wsp *= 606 nucleotides; *16S* rRNA = 374 nucleotides) were exported to MEGA 7.0 software for further alignment and analysis using Clustal W algorithm [63]. Phylogenetic tree for the *wsp* gene was constructed using the Maximum Likelihood (best-fit substitution model) method. The phylogenetic tree model with lowest Bayesian information criterion (BIC) is considered to describe the substitution pattern the best. Hence, Tamura 3-parameter model was chosen for *wsp* gene analysis. *Wsp* gene sequence from *Brugia pahangi* was incorporated as the outgroup to confirm the outcome of the phylogenetic tree. For *16S* rRNA gene, the evolutionary history was inferred using the Maximum Likelihood method (best-fit substitution model). Based on the lowest BIC value, Jukes-Cantor model was chosen to infer the phylogenetic relationship of *Wolbachia* using the *16S* rRNA gene. The *16S* rRNA gene sequence of *Rickettsia japonica* was included as the outgroup. Both phylogeny tests were performed by bootstrap method with 1000 replications. All the evolutionary analyses were performed in MEGA 7.0 software. All sequences used in the phylogenetic analyses were submitted in the GenBank database under the accession numbers MN893348-MN893366 (*wsp* gene) and MN887537-MN887588 (*16S* rRNA).

## Results

### Detection of *Wolbachia* in mosquitoes

A total of 217 adult mosquitoes representing 26 mosquito species belonging to 5 genera (*Anopheles*, *Culex*, *Aedes, Mansonia* and *Armigeres*) (Additional file 1: Table S1) were screened for *Wolbachia.* These mosquitoes were collected from a wide range of habitats such as urban, island and forest (Additional file 1: Table S2). Polymerase chain reaction amplifying the *Wolbachia wsp* gene was successful in most of the Culicinae mosquito samples except for *Culex vishnui*, *Culex pseudovishnui*, *Mansonia* mosquitoes and some samples of *Culex quinquefasciatus. Wolbachia* infection was detected in these samples as well as in *Anopheles* spp. mosquitoes using the *16S* rRNA primers.

The overall *Wolbachia* infection rate for all mosquitoes tested in this study was 46.1%. *Wolbachia* was detected in 4 species of mosquitoes (40/217) using *wsp* gene amplification and 15 species of mosquitoes (60/217) using *16S* rRNA gene amplification (Table 1). The prevalence of *Wolbachia* infection within mosquito species ranged from 35.3% (6/17) in *Cx. quinquefasciatus* to 100% (19/19) in *Ae. albopictus* for *wsp* gene. *Wolbachia* infection rate by detection of *Wolbachia 16S* rRNA gene ranged between 50–100% for *Cx. sinensis*, *Cx. vishnui*, *Cx. pseudovishnui* and *Mansonia* mosquitoes. For *Anopheles* mosquitoes, the infection rate of *Wolbachia* among each species ranged between 21–57.1%.

**Table 1:** *Wolbachia* infection prevalence rates in adult female mosquitoes using two conserved *Wolbachia* genes (*16S* rRNA and *wsp*)

Mosquito species	*n*	PCR-positive		Prevalence (95% CI) (%)^a^	
		*wsp*	*16S* rRNA	*wsp*	*16S* rRNA
*Anopheles* (*Cellia*) group	1	0	0	0	0
*An. balabacensis*	19	0	4	0	21.1 (7.0–46.1)
*An. introlatus*	54	0	17	0	31.5 (20.0–45.7)
*An. macarthuri*	4	0	1	0	25.0 (1.3–78.1)
*An. latens*	8	0	4	0	50.0 (17.5–82.6)
*An. karwari*	2	0	0	0	0
*An. kochi*	1	0	0	0	0
*An. maculatus*	9	0	2	0	22.2 (3.9–59.8)
*An. vagus*	3	0	0	0	0
*An. watsonii*	5	0	0	0	0
*An. barbirostris*	10	0	5	0	50.0 (20.1–79.9)
*An. umbrosus*	6	0	0	0	0
*An. hyrcanus*	18	0	9	0	50.0 (26.8–73.2)
*An. sinensis*	7	0	4	0	57.1 (20.2–88.2)
*An. lindesayi* species group	1	0	0	0	0
*An. separatus*	1	0	0	0	0
*An. aconitus*	1	0	0	0	0
*Cx. vishnui*	1	0	1	0	100 (5.5–100)
*Cx. pseudovishnui*	2	0	2	0	100 (19.8–100)
*Cx. quinquefasciatus*	17	6	5	35.3 (15.3–61.4)	29.4 (11.4–56.0)
*Cx. sinensis*	2	0	1	0	50.0 (2.7–97.3)
*Ma. annulifera*	1	0	1	0	100 (5.5–100)
*Ma. bonneae*	1	0	1	0	100 (5.5–100)
*Ar. subalbatus*	22	13	0	59.1 (36.7–78.5)	0
*Ae. aegypti*	2	2	0	100 (19.8–100)	0
*Ae. albopictus*	19	19	3	100 (79.1–100)	15.8 (4.2–40.5)

*Note*: An individual was considered *Wolbachia-*infected when any one of the two *Wolbachia* gene fragments were amplified

*Abbreviation*: CI, confidence interval

^a^*Wolbachia* prevalence rate (%) = [No. of *Wolbachia* positive mosquitoes/Total no. of mosquitoes] × 100

**Fig. 2:**
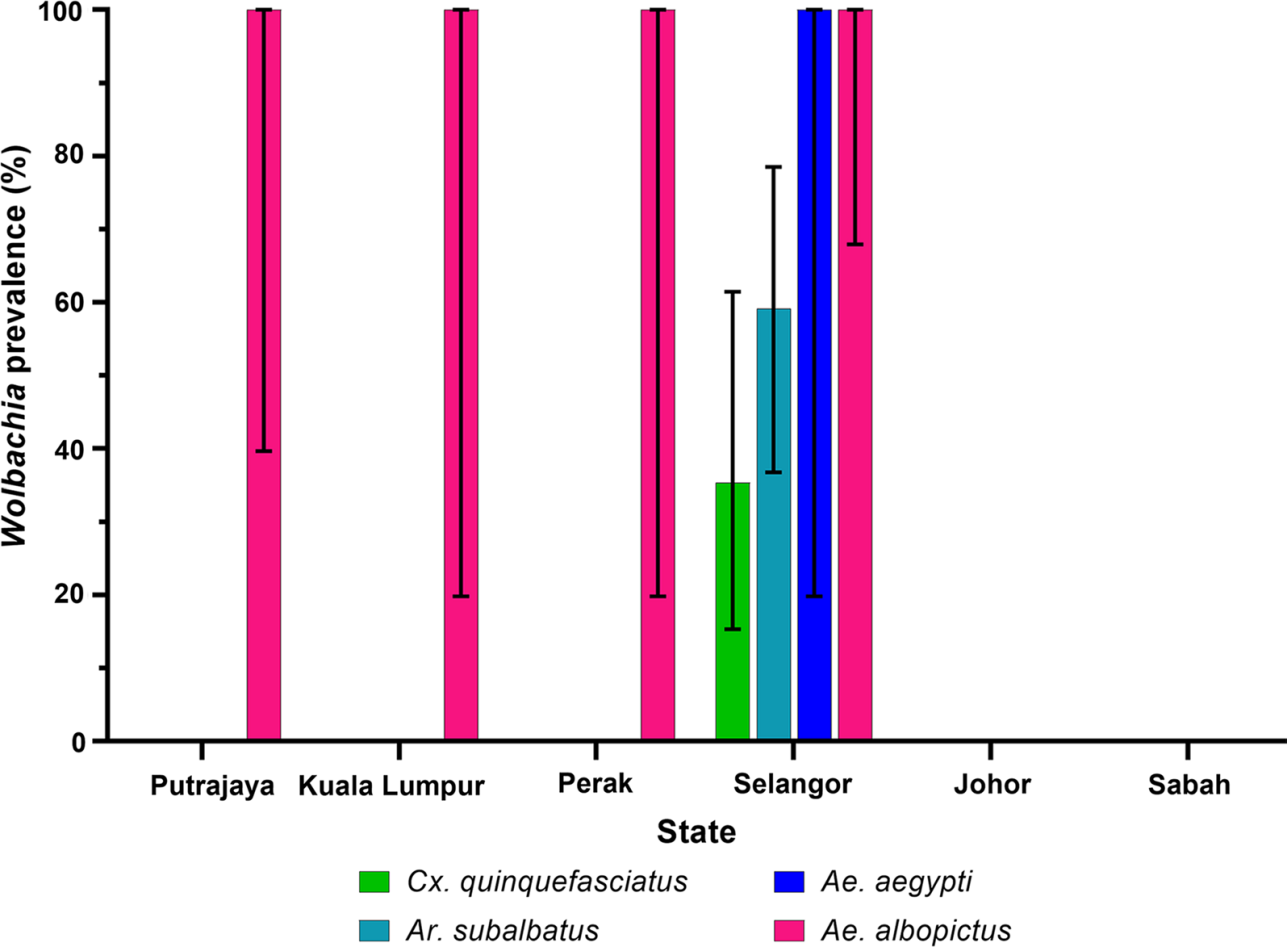
Prevalence of *Wolbachia*-infected mosquitoes collected from different states in Malaysia using *wsp* primers. Error bar denotes 95% confidence interval (CI)

**Fig. 3:**
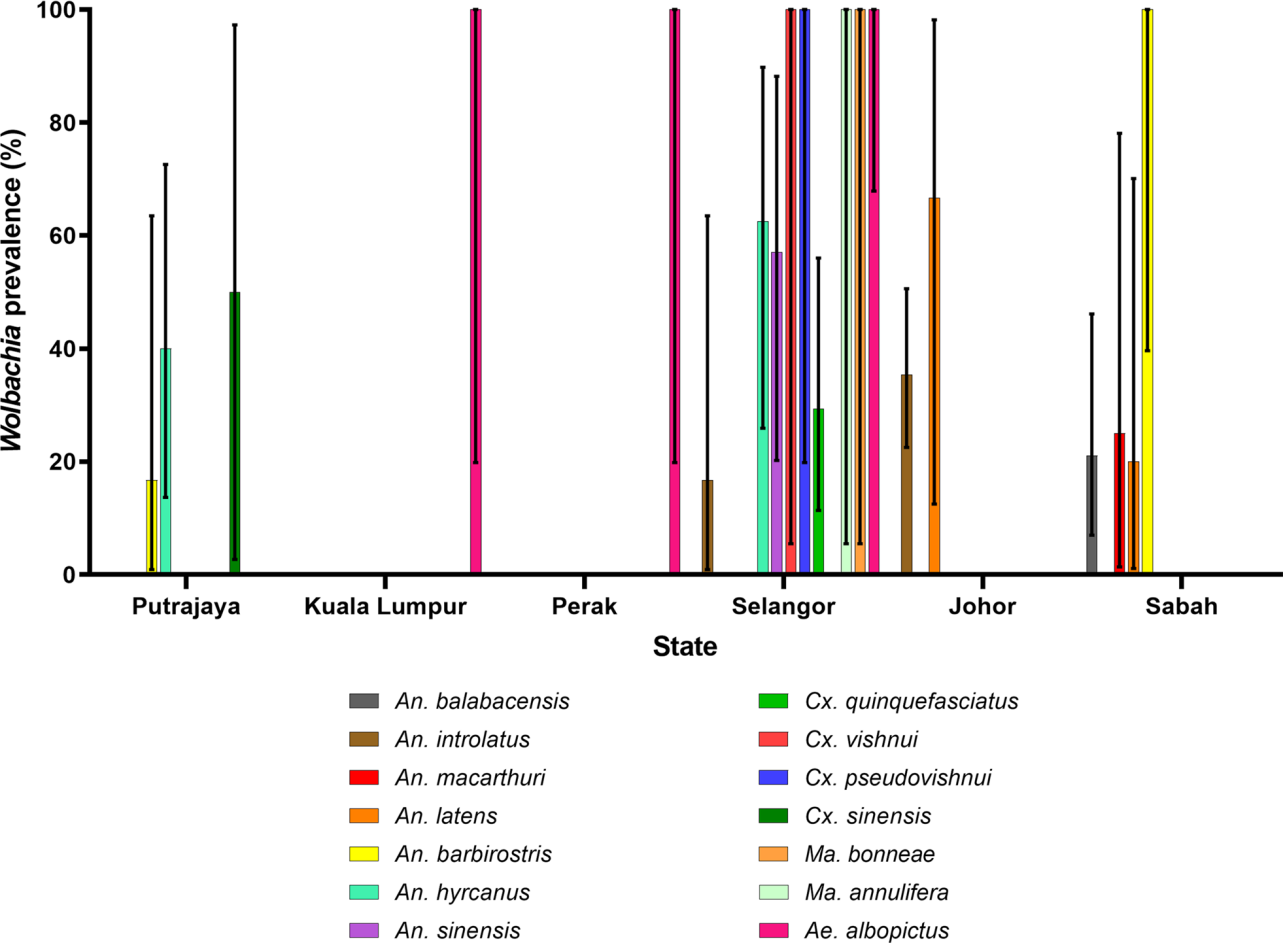
Prevalence of *Wolbachia*-infected mosquitoes collected from different states in Malaysia using *16S* rRNA primers. Error bar denotes 95% confidence interval (CI)

Presence of *Wolbachia* in different mosquito species from each state is given in Fig. 2 (*wsp* gene) and Fig. 3 (*16S* rRNA). While the prevalence appeared to be higher in certain states such as Selangor, this bias could be caused by the prevalence of *Wolbachia* in *Ae. albopictus* or higher number of mosquito species collected from Selangor (Fig. 3). Thus, it would be more valid if comparisons were made among mosquito species found in more than one site and are available in similar numbers, without taking *Ae. albopictus* into account. For example, the prevalence of *Wolbachi*a was higher in *An. barbirostris* from Sabah than from Putrajaya, and the prevalence of *Wolbachia* was also higher in *An. hyrcanus* from Selangor than from Putrajaya. Similarly, the same scenario applies to the prevalence of *Wolbachia* in the mosquitoes according to ecological types (Table 2), as most mosquitoes were collected from forested areas. Nonetheless, none of the *An. balabacensis* from the Banggi Island was found to be infected with *Wolbachia*, compared to infections found in the same species from mainland forested area. As for *An. hyrcanus*, 40% of the mosquitoes from the wetland were infected compared to 50–75% from the forested area. Thus, mosquitoes from forested areas, may have higher prevalence of *Wolbachia* compared to those from the wetland (Putrajaya) or island (Banggi Island) areas.

**Table 2:** *Wolbachia* infection prevalence rates in adult female mosquitoes in different ecological types across the states in Malaysia

Ecological type/study site	Mosquito species	Prevalence (95% CI) (%)	
		*wsp*	*16S* rRNA
Urban			
Bangsar, Kuala Lumpur	*Ae. albopictus*	100 (5.5–100)	–
Jalan Genting, Kuala Lumpur		100 (5.5–100)	100 (5.5–100)
Pasir Puteh, Perak		100 (19.8–100	100 (19.8–100)
Damansara Damai, Selangor	*Ae. aegypti*	100 (19.8–100)	–
Persanda, Selangor	*Cx. quinquefasciatus*	42.9 (11.8–79.8)	28.6 (5.1–69.8)
Klang, Selangor		30.0 (8.1–64.6)	30.0 (8.1–64.6)
Island			
Pulau Ketam, Selangor	*Ae. albopictus*	100 (39.6–100)	–
Pulau Banggi, Sabah	*An. balabacensis*	0	0
Wetland			
Putrajaya	*An. hyrcanus*	0	40.0 (13.7–72.6)
	*An.* (*Cellia*) group	0	0
	*An. barbirostris*	0	20.0 (1.1–70.1)
	*An. umbrosus*	0	0
	*An. maculatus*	0	0
	*An. karwari*	0	0
	*An. separatus*	0	0
	*An. lindesayi* group	0	0
	*Cx. sinensis*	0	50.0 (2.7–97.3)
	*Ae. albopictus*	100	–
Forest			
Ulu Kalong, Selangor	*An. introlatus*	0	16.7 (0.9–63.5)
	*An. maculatus*	0	100 (19.8–100)
	*Ar. subalbatus*	59.1 (36.7–78.5)	–
Ulu Kalong, Selangor	*Ma. bonneae*	0	100 (5.5–100)
	*Ae. albopictus*	100 (56.1–100)	–
	*Culex.* sp.	100 (5.5–100)	–
Bukit Lagong, Selangor	*An. maculatus*	0	0
	*An. hyrcanus*	0	75.0 (21.9–98.7)
	*Ma. annulifera*	0	100 (5.5–100)
	*Cx. vishnui*	0	100 (5.5–100)
	*Cx. pseudovishnui*	0	100 (5.5–100)
Sg. Sendat, Selangor	*An. sinensis*	0	57.1 (20.2–88.2)
	*An. hyrcanus*	0	50.0 (9.2–90.8)
Tawau, Sabah	*An. vagus*	0	0
	*An. maculatus*	0	0
	*An. watsonii*	0	0
	*An. funestus*	0	0
	*An. kochi*	0	0
	*An. macarthuri*	0	25.0 (1.3–78.1)
	*An. latens*	0	20.0 (1.1–70.1)
	*An. balabacensis*	0	23.5 (7.8–50.2)
	*An. barbirostris*	0	100 (39.6–100)
Kluang, Johor	*An. introlatus*	0	55.6 (22.7–84.7)
Mersing, Johor	*An. introlatus*	0	24.2 (11.7–42.6)
	*An. latens*	0	100 (5.5–100)
Kota Tinggi, Johor	*An. introlatus*	0	50.0 (14.0–86.1)
	*An. latens*	0	50.0 (2.7–97.3)
	*An. umbrosus*	0	0

*Abbreviation*: CI, confidence interval

### *Plasmodium-*positive *Anopheles* with *Wolbachia* infection

Out of the 34 *Plasmodium*-positive *Anopheles* samples, 14 (38.24%) were also found to have *Wolbachia* infection (Table 3). Interestingly, only 1 out of 9 (11.11%) samples with oocysts/*Plasmodium* DNA-positive abdomens were positive for *Wolbachia*, as compared to 4 out of 8 (50%) samples with sporozoites/*Plasmodium* DNA-positive head/thorax which were also *Wolbachia*-infected. However, this difference was not significant (Fisher’s exact test, two-tailed, *P* = 0.131). There was also no particular difference in *Wolbachia* prevalence between the small number of *Plasmodium*-positive *Anopheles* species investigated in this study.

**Table 3:** *Plasmodium-*positive *Anopheles* with *Wolbachia* infection

Species	No. of samples	No. of samples: stage/type of infection	No. of *Wolbachia*-positive samples/No. of samples	No. of samples positive for *Wolbachia*/Total no. of samples
*An. introlatus*	28	17: *Plasmodium* DNA-positive whole mosquito; 4: oocysts/*Plasmodium* DNA-positive abdomen; 7: sporozoites/*Plasmodium* DNA-positive head/thorax	8/17 whole mosquito samples; 0/4 samples with oocysts/*Plasmodium* DNA-positive abdomen; 4/7 samples with sporozoites/*Plasmodium* DNA-positive head/thorax	12/28
*An. latens*	2	1: oocysts; 1: *Plasmodium* DNA-positive abdomen	1/1 sample with oocysts; 0/1 sample with *Plasmodium* DNA-positive abdomen	1/2
*An. balabacensis*	2	1: sporozoites; 1: oocysts	0	0/2
*An. umbrosus*	2	2: oocysts	0	0/2
Total	34	17: whole mosquitoes; 9: oocysts/*Plasmodium* DNA-positive abdomen 8: sporozoites/*Plasmodium* DNA-positive head/thorax	8/17 whole mosquito samples; 1/9 samples with oocysts/*Plasmodium* DNA-positive abdomen; 4/8 samples with sporozoites/*Plasmodium* DNA-positive head/thorax	13/34

### Phylogenetic analysis of *Wolbachia* in mosquitoes

**Fig. 4:**
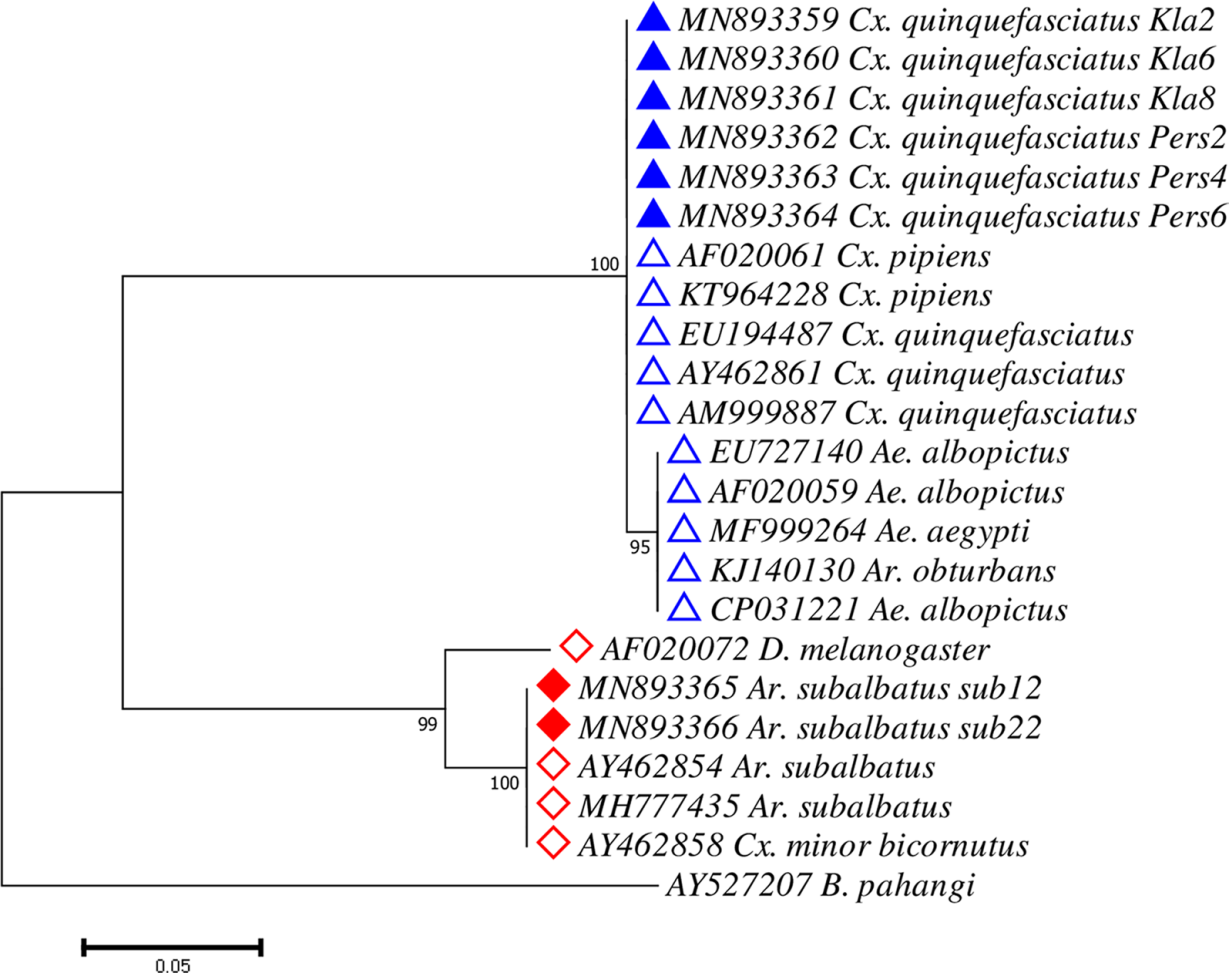
Maximum Likelihood phylogenetic analysis for *Wolbachia* using the *wsp* gene. The tree with the lowest Bayesian Information Criterion (BIC) (2725.733879) is shown, incorporating 33 nucleotide sequences (8 sequences from this study; 14 sequences from GenBank) and one outgroup (*B. pahangi*). *Wolbachia* sequences of Supergroup A are denoted with red diamonds while those of Supergroup B are denoted with blue triangles. Sequence retrieved from GenBank is denoted with hollow diamond or triangle along with its respective accession number while sequence obtained from this study are denoted with a filled diamond or triangle

Eight *wsp Wolbachia* sequences from this study were deemed suitable for phylogenetic analysis after analysis of the sequencing results. We retrieved an additional 14 *wsp* sequences and 1 outgroup sequence (*B. pahangi*) from GenBank for phylogenetic analysis. In total, 23 sequences were used for construction of the phylogenetic tree. The alignments for different species of mosquitoes are shown in Additional file 2: Figure S1 (Supergroup A) and Additional file 3: Figure S2 (Supergroup B). Phylogenetic tree of *wsp* gene inferred using ML method based on Tamura 3-parameter model (lowest BIC value = 2502.136) resulted in two major clades belonging to Supergroups A and B with bootstrap values of 99% and 100%, respectively (Fig. 4). Within the *Wolbachia* Supergroup B, two sub-clades branched out, one comprised of *w*AlbB sequences from *Ae. albopictus* and *Ae. aegypti* (bootstrap value = 95%) whereas *Wolbachia* from *Cx. quinquefasciatus* were grouped with *w*Pip from *Cx. pipiens* (bootstrap value = 100%).

**Fig. 5:**
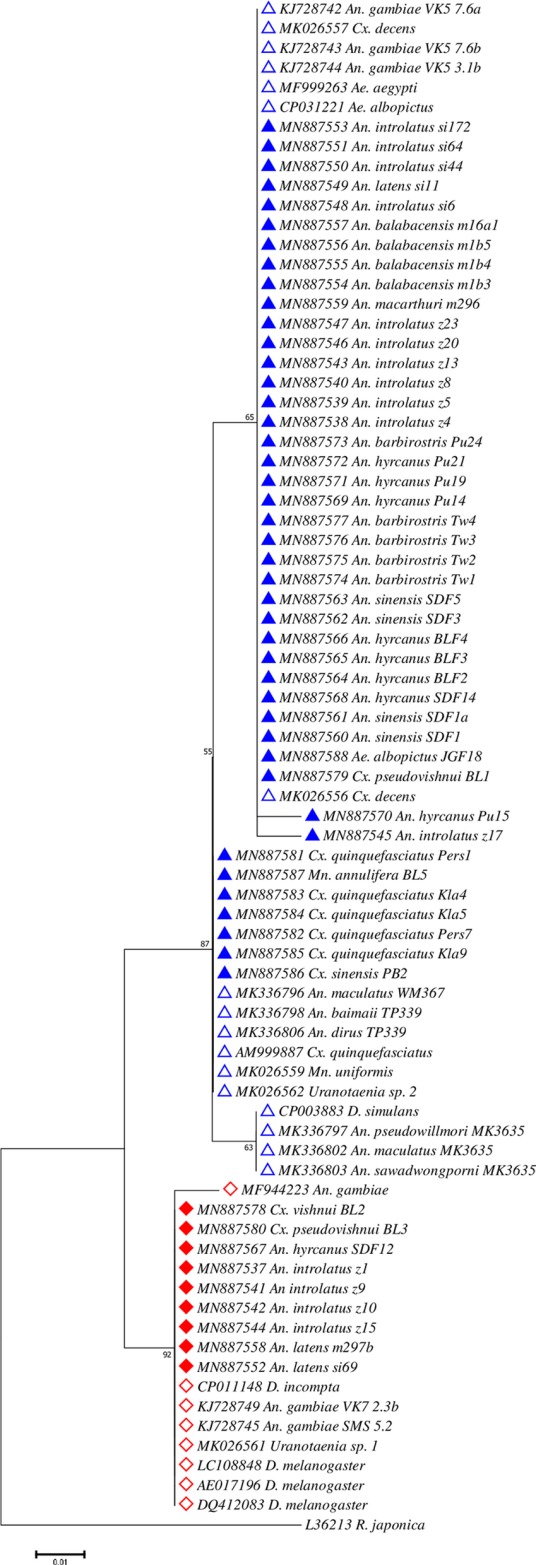
Maximum Likelihood phylogenetic analysis for *Wolbachia* using the *16S* rRNA gene. The tree with the lowest Bayesian Information Criterion (BIC) (2020.770483) is shown, incorporating 83 nucleotide sequences (52 sequences from this study; 31 sequences from GenBank) and one outgroup (*R. japonica*). *Wolbachia* sequences of Supergroup A are denoted with red diamonds while those of Supergroup B are denoted with blue triangles. Sequences retrieved from GenBank are denoted with an open diamond or triangle along with its respective accession number while sequences obtained from this study are denoted with a filled diamond or triangle

A total of 52 *16S* rRNA *Wolbachia* sequences were deemed suitable for use in phylogenetic analysis after analysing the sequencing results. Confirmation of *Wolbachia* supergroup for each sequence was executed using BLAST. All the sequences showed 99–100% nucleotide identities. Additionally, 26 sequences from GenBank were retrieved for phylogenetic analysis including 1 outgroup sequence (*R. japonica*). Overall, 84 sequences were used for construction of *Wolbachia 16S* rRNA phylogenetic tree. The alignments of *16S* rRNA sequences are shown in Additional file 4: Figure S3 (Supergroup A) and Additional file 5: Figure S4 (Supergroup B). The phylogenetic tree inferred using Maximum Likelihood method based on Jukes-Cantor model (lowest BIC value = 1897.45) resulted in two major clades with high bootstrap value: Supergroup A (bootstrap value = 91%) and Supergroup B (bootstrap value = 85%) (Fig. 5). Within the Supergroup B clade, three sub-clades branched out. The first sub-clade consists of the majority of the *Wolbachia* sequences obtained from *Anopheles* mosquitoes (82.9%) from this study and *Wolbachia* sequences from *An. gambiae*, *Ae. albopictus* and *Ae. aegypti* retrieved from GenBank (Fig. 5). *Wolbachia 16S* rRNA gene amplified from adults of *Cx. quinquefasciatus* were clustered within the second sub-clade of *Wolbachia* Supergroup B, confirming the phylogenetic relationship inferred using *wsp* gene. A single sample of each *Ma. annulifera* and *Ma. bonneae* was found infected with *Wolbachia* using *16S* rRNA gene. Only *Wolbachia*-infected *Ma. annulifera* sequence was obtained in this study and it was grouped together in the Supergroup B with *w*Unif-Mad isolated from *Ma. uniformis*, as well as *Wolbachia* from *Cx. quinquefasciatus* and *Anopheles* mosquitoes from Kayin state, Myanmar. Repeated attempts to sequence *Wolbachia 16s* rRNA gene from *Wolbachia*-infected *Ma. bonneae* failed to provide reliable sequences and thus were not available. The third sub-clade comprised *Wolbachia* sequences from *Anopheles* mosquitoes found in Myanmar. On the other hand, *Wolbachia* from two Culicinae and seven *Anopheles* spp. mosquitoes were grouped with *Wolbachia* Supergroup A.

While investigating if any strains of *Wolbachia* was associated with a particular species of mosquito, *Wolbachia* from *Armigeres subalbatus* and the only one sample from *Cx. vishnui* were all found to be grouped in Supergroup A, whereas *Wolbachia* from *Cx. quinquefasciatus* mosquitoes were of Supergroup B. Other than these, *Wolbachia* from other mosquito species belonged to either supergroups, although most of them were grouped in *Wolbachia* Supergroup B.

## Discussion

In our study, we assessed the prevalence of *Wolbachia* in mosquito species collected from Peninsular Malaysia and Sabah. To our knowledge, this study is the first report of natural *Wolbachia* infections in field adult mosquito populations in Malaysia detected using *Wolbachia wsp* and *16S* rRNA PCR amplifications. Our study further supports the higher sensitivity of the *16S* rRNA nested PCR described by Shaw et al. [39] over the standard *wsp* PCR from Zhou et al. [61] for the detection of *Wolbachia* in mosquitoes due to genetic divergence or weak infection density [44]. The overall infection rate of all tested mosquitoes in this study was 46.1%. *Wolbachia* infection rates of 28.1% and 37.8% have been previously reported in the natural mosquito populations from neighboring countries Singapore [3] and Thailand [8], respectively.

The nested PCR utilizing *16S* rRNA primers detected *Wolbachia* in seven species of *Anopheles* (41/144; 28.5%), i.e. *An. balabacensis*, *An. latens*, *An. introlatus*, *An. macarthuri* (Leucosphyrus Group), *An. barbirostris* (Barbirostris Group), *An. hyrcanus* and *An. sinensis* (Hyrcanus Group). Of these, *An. latens* [64], *An. introlatus* [59] and *An. balabacensis* [65] of the Leucosphyrus Group were incriminated as vectors of *Plasmodium knowlesi* in Malaysia. Additionally, *An. balabacensis* is also a vector of human malaria and Bancroftian filariasis in Sabah, Malaysia [66]. Other *Anopheles* species such as *An. barbirostris* [67] and *An. sinensis* [68] are vectors of human malaria, whereas *An. hyrcanus* has been postulated to be a vector for malaria due to its simultaneous occurrence with malaria cases [69]. These data along with the report of *Wolbachia* in natural mosquito populations in Kayin state, Myanmar [46] and Africa [38–40, 42–44], reinforce the occurrence of natural *Wolbachia* infections in malaria vectors.

Conclusive prevalence of *Wolbachia* based on different habitats cannot be drawn. This is due to the heterologous distribution of the mosquito species themselves and differences in number of mosquito species available between the sites. The prevalence appeared to be biased in certain states or environments mostly due to more mosquito species collected from the particular site than the others. However, there was a slight indication that *Wolbachia* infections can be more prevalent in forested areas than wetlands or island. This can be attributed to the richer flora and fauna in the forested areas which may have many more hosts with stable *Wolbachia*, which may facilitate horizontal transfers to other species.

Molecular phylogeny based on *16S* rRNA sequences revealed that most *Wolbachia* infecting *Anopheles* (34/41) clustered with *w*AlbB (Supergroup B) isolated from *Ae. albopictus* [70], whereas the remaining *Wolbachia* strains from other *Anopheles* (7/41) were more closely related to *w*Mel (*D. melanogaster*) of Supergroup A. Interestingly, all the *Wolbachia* infecting *Anopheles* detected in this study were shown to be more closely related with those recently reported in African malaria vectors (*w*Anga) [38–40, 42–44] than with the ones from Myanmar [46] (Fig. 5). Although recent publications suggest a lack of concrete evidence regarding the presence of *Wolbachia* in *Anopheles* and *Ae. aegypti* [71, 72], our results show that *Wolbachia* is present in *Anopheles.* Our sequences were clean and matched those of *Wolbachia* Supergroup A and B. However, we agree that our limited study shows that the prevalence of *Wolbachia* in *Anopheles* is much lower than the natural presence of *Wolbachia* in *Ae. albopictus*.

*Plasmodium*-infected *Anopheles* mosquitoes were also found to be *Wolbachia*-infected. These mosquitoes harbored oocysts or sporozoites. Results from this study found that *Wolbachia* prevalence is higher (though not significant) in *Anopheles* mosquitoes with sporozoites (50%) than in mosquitoes with oocysts (11.11%). This is in agreement with a study finding significant reductions in *P. berghei* or *P. falciparum* oocyst infections in *An. gambiae* transiently and somatically-infected with *w*MelPop or *w*AlbB *Wolbachia* strains [36, 37]. It was also found that natural populations of *Wolbachia*-infected *An. coluzzii* females have lower frequency of *Plasmodium* infections than *Wolbachia*-negative individuals. Although the study did not explicitly mention the infection stages of the parasite in these mosquitoes, it is assumed that most of these *Plasmodium* infections could be at the oocyst stage since *Plasmodium* DNA was detected from dissected abdomens and thoraces of the mosquitoes 5 days after indoor collection [39]. Another interesting study also showed that *Culex pipiens* (the natural vector of *P. relictum*), naturally infected with *Wolbachia*, had increased sporozoites than those without *Wolbachia* [73]. Thus, it seems to demonstrate that when *Wolbachia* is naturally present in the mosquitoes, it may perturb oocysts development but promote sporozoites production. This however, is the opposite to the findings of a study on *An. gambiae* from Mali, Africa showing that *Wolbachia* infection was significantly lower in sporozoite-positive mosquitoes compared to the sporozoite-negative mosquitoes. However, *Wolbachia*-positive mosquitoes had higher oocyst infection compared to *Wolbachia* negative-mosquitoes [40]. It seems that *Wolbachia* can suppress the prevalence and intensity of *P. falciparum* sporozoite infections in wild *An. gambiae* [40]. On the other hand, *An. stephensi* experimentally infected with *w*AlbB strain of *Wolbachia* was able to suppress both *P. falciparum* oocysts and sporozoite infection rates [34]. Due to these contrasting results, it is pertinent for us to study the life-cycle and transmission dynamics of *Plasmodium* in *Anopheles* vectors infected and uninfected with *Wolbachia.* It is possible that *Wolbachia* may affect only certain *Plasmodium* parasites present in the vector such as *P. falciparum* as reported by Bian et al. [34] or simian malaria parasites in this study, or it might influence vector competence of certain vector species [73]. As such, effect of *Wolbachia* on *An. balabacensis*, *An. latens* and *An. introlatus* in the present study remains obscure and hence, there is a need for extensive mosquito collections and thorough investigations.

Naturally, *Ae. aegypti* does not harbor *Wolbachia*. Interestingly, in this study the two *Ae. aegypti* which emerged from larvae collected from sticky traps were positive for *Wolbachia*. However, *Wolbachia* has been found in large percentage of *Ae. aegypti* in Manila [47], and in Kuala Lumpur, Malaysia, *Wolbachia* of Supergroup B has been reported from *Ae. aegypti* larvae which were also positive for DENV 2 virus [49]. Similarly, in India [50], *Wolbachia* has been detected in natural *Ae. aegypti* populations and was designated as *w*AegB. Additionally, *Wolbachia*-infected *Ae. aegypti* has been reported from Mexico (> 50%) [52], Florida [51], Panama [54] and Texas [53]. It is noteworthy that during this study, no *Wolbachia*-infected mosquito colony is being reared in our insectarium, neither is there any routine screening nor handling of *Wolbachia* material.

The *Wolbachia* infecting *Cx. quinquefasciatus* was shown to be more closely related to *w*Pip, isolated from *Cx. pipiens* within the Supergroup B than with *w*AlbB from *Ae. albopictus*, similar to previous reports [4, 74]. Our results also revealed the presence of *Wolbachia* in *Ar. subalbatus* which was grouped within the Supergroup A. Interestingly, all *Cx. vishnui*, *Cx. pseudovishnui*, *Cx. sinensis* (vector of Japanese encephalitis), *Ma. annulifera*, *Ma. bonneae* and some *Cx. quinquefasciatus* mosquitoes failed to show a positive *wsp* PCR amplification. Through nested PCR of the *16S* rRNA gene, this is the first report of *Wolbachia* infection detected in *Cx. sinensis*, *Cx. vishnui*, *Cx. pseudovishnui*, *Ma. bonneae* and *Ma. annulifera*. However, due to the low number of samples collected, further studies are needed to determine if this is a result of strain variability or low-density infections which resulted in failure of amplification using *wsp* primers.

While the PCR and sequencing results of the present study are convincing, especially by showing that the *Wolbachia* strains found infecting the mosquitoes belong to the two supergroups (A and B, which are associated with arthropods), there is a growing call for careful investigations into natural *Wolbachia* infections in previously unreported mosquitoes. This is because, it has been estimated that over half of terrestrial arthropod species are infected with *Wolbachia* [75]. Therefore, the environment is a factor that possibly affects the presence or absence of *Wolbachia* in some mosquitoes [43]. Thus, environmental contamination of the samples from plants, endo- and ectoparasites, *Wolbachia*-contaminated/infected food in the wild, eggs and larval habitats and other cohabitating insects cannot be ruled out [46, 71]. Furthermore, reports of *Wolbachia* found with low prevalence in some species and difficulty in detecting them, especially in *Anopheles* spp. [42–44, 46], indicate that there could be a lack of stable symbiotic relationship between *Wolbachia* and the hosts [71]. Therefore, to demonstrate evidence of a stable, intraovarially-transmitted *Wolbachia* symbiont in a host [71], steps that should be taken include: (i) visualizing *Wolbachia* in different host tissues using fluorescent *in situ* hybridization (FISH) or electron microscopy; (ii) demonstrating that the infection is and can be maternally transmitted by performing reciprocal crosses; and (iii) showing that *Wolbachia* can be removed from the mosquitoes by antibiotic or heat treatment [72].

Nonetheless, the growing reports of *Wolbachia* infections in previously unreported mosquito vectors, open avenues for further investigations into its prevalence and should prompt careful evaluations on the release of *Wolbachia*-infected mosquitoes for disease/vector control programmes. Additionally, the presence of *Asaia*, an acetic acid bacterium found in some *Anopheles* species is postulated for the absence of *Wolbachia* in *Anopheles* mosquitoes [76]. *Asaia* has been shown to impede vertical transmission of *Wolbachia* in *Anopheles* mosquitoes experimentally and found negatively related with *Wolbachia* in mosquito reproductive organs [77]. Therefore, the dynamics of *Wolbachia* infections with vector capacity, microbial interactions within the host and disease transmission require further in-depth studies.

## Conclusions

The present study reports the presence of *Wolbachia* in *Anopheles* and *Ae. aegypti* which were not previously reported, as well as in other important mosquito vector genera such as *Culex* and *Mansonia* using *16S* rRNA and *wsp* genes. Given the fact that *Wolbachia* can reduce the life span of its host, prevent pathogen from completing its life-cycle, as well as reduce susceptibility of host to pathogen infection, *Wolbachia* is being considered for vector control programmes and is now released on a large scale in many countries. Thus, it is pertinent to carry out large-scale studies on the natural infection of *Wolbachia* in mosquito vectors using more sensitive and accurate methods of detection as described above. The long-term effect of introduced *Wolbachia* into new hosts and its effect on pathogen suppression should also be studied.

## Supplementary information

**Additional file 1: Table S1.** Summary of mosquito species used in the study from six states in Malaysia. Collection was carried out using human landing catch (HLC), mosquito magnetic trap and ovitrap. **Table S2.** Mosquito collection sites. Mosquitoes were collected from different settings: urban, village, island, forest and wetland across several states in Malaysia. **Table S3.** *Anopheles* samples with *Plasmodium* infections.

**Additional file 2: Figure S1.** Alignment and variable sites of *Wolbachia wsp* sequences (Supergroup A) with nucleotide positions.

**Additional file 3: Figure S2.** Alignment and variable sites of *Wolbachia wsp* sequences (Supergroup B) with nucleotide positions.

**Additional file 4: Figure S3.** Alignment and variable sites of *Wolbachia 16S* rRNA (Supergroup A) sequences with nucleotide positions.

**Additional file 5: Figure S4.** Alignment and variable sites of *Wolbachia 16S* rRNA (Supergroup B) sequences with nucleotide positions.

## Data Availability

All data generated or analysed during this study are included in this published article and its additional files. The newly generated sequences were deposited in the GenBank database under the accession numbers MN893348-MN893366 (*wsp* gene) and MN887537-MN887588 (*16S* rRNA).

## Abbreviations

*wsp*: *Wolbachia* surface protein
*16S* rRNA: *16S* ribosomal RNA
