# Supplementary material for: Natural Wolbachia infection in field-collected Anopheles and other mosquito species from Malaysia
Source: Parasit Vectors. 2020 Aug 12;13:414. doi: 10.1186/s13071-020-04277-x (PMC7425011; doi:10.1186/s13071-020-04277-x)
Supplement: Supplementary file 1 — Additional file 1: Table S1. Summary of mosquito species used in the study from six states in Malaysia. Collection was carried out using human landing catch (HLC), mosquito magnetic trap and ovitrap. Table S2. Mosquito collection sites. Mosquitoes were collected from different settings: urban, village, island, forest and wetland across several states in Malaysia. Table S3. Anopheles samples with Plasmodium infections. [file 13071_2020_4277_MOESM1_ESM.docx]

**Additional file 1: Table S1.** Summary of mosquito species used in the study from six states in Malaysia. Collection was carried out using human landing catch (HLC), mosquito magnetic trap and ovitrap.

| **Mosquito species** | **State** | | | | | | **Total** |
| --- | --- | --- | --- | --- | --- | --- | --- |
|  | **Putrajaya** | **Kuala Lumpur** | **Perak** | **Selangor** | **Johor** | **Sabah** |  |
| *Anopheles* (*Cellia*) group | 1 | 0 | 0 | 0 | 0 | 0 | **1** |
| *An. balabacensis* | 0 | 0 | 0 | 0 | 0 | 19 | **19** |
| *An. introlatus* | 0 | 0 | 0 | 6 | 48 | 0 | **54** |
| *An. macarthuri* | 0 | 0 | 0 | 0 | 0 | 4 | **4** |
| *An. latens* | 0 | 0 | 0 | 0 | 3 | 5 | **8** |
| *An. karwari* | 2 | 0 | 0 | 0 | 0 | 0 | **2** |
| *An. kochi* | 0 | 0 | 0 | 0 | 0 | 1 | **1** |
| *An. maculatus* | 1 | 0 | 0 | 3 | 0 | 5 | **9** |
| *An. vagus* | 0 | 0 | 0 | 0 | 0 | 3 | **3** |
| *An. watsonii* | 0 | 0 | 0 | 0 | 0 | 5 | **5** |
| *An. barbirostris* | 6 | 0 | 0 | 0 | 0 | 4 | **10** |
| *An. umbrosus* | 2 | 0 | 0 | 0 | 4 | 0 | **6** |
| *An. hyrcanus* | 10 | 0 | 0 | 8 | 0 | 0 | **18** |
| *An. sinensis* | 0 | 0 | 0 | 7 | 0 | 0 | **7** |
| *An.lindesayi* species group | 1 | 0 | 0 | 0 | 0 | 0 | **1** |
| *An. separatus* | 1 | 0 | 0 | 0 | 0 | 0 | **1** |
| *An. aconitus* | 0 | 0 | 0 | 0 | 0 | 1 | **1** |
| *Cx. vishnui* | 0 | 0 | 0 | 1 | 0 | 0 | **1** |
| *Cx.pseudovishnui* | 0 | 0 | 0 | 2 | 0 | 0 | **2** |
| *Cx. quinquefasciatus* | 0 | 0 | 0 | 17 | 0 | 0 | **17** |
| *Cx. sinensis* | 2 | 0 | 0 | 0 | 0 | 0 | **2** |
| *Ma. annulifera* | 0 | 0 | 0 | 1 | 0 | 0 | **1** |
| *Ma. bonneae* | 0 | 0 | 0 | 1 | 0 | 0 | **1** |
| *Ar. subalbatus* | 0 | 0 | 0 | 22 | 0 | 0 | **22** |
| *Ae. aegypti* | 0 | 0 | 0 | 2 | 0 | 0 | **2** |
| *Ae.albopictus* | 4 | 2 | 2 | 11 | 0 | 0 | **19** |
| **Total** | **30** | **2** | **2** | **81** | **55** | **47** | **217** |

**Additional file 1: Table S2.** Mosquito collection sites. Mosquitoes were collected from different settings: urban, village, island, forest and wetland across several states in Malaysia.

| Site | State | GPS coordinates | Environment |  |
| --- | --- | --- | --- | --- |
| Pasir Puteh | Perak | N 4°34'31.2", E 101°04'35.4" | Urban |  |
| Pulau Ketam | Selangor | N 3°01'18.5", E 101°15'12.4" | Island |  |
| Sunway | Selangor | N 3°04'54.3", E 101°36'38.6" | Urban |  |
| Damansara Damai | Selangor | N 3°11'34.8", E 101°35'32.3" | Urban |  |
| Persanda | Selangor | N 3°04'32.9", E 101°33'11.9" | Urban |  |
| Sungai Sendat | Selangor | N 3°25'03.3", E 101°39'58.4" | Forest |  |
| Ulu Kalong | Selangor | N 3°25'09.7", E 101°40'27.2" | Forest |  |
| Bukit Lagong | Selangor | N 3°15'43.8", E 101°37'31.3" | Forest |  |
| Bangsar | Federal Territory of Kuala Lumpur | N 3°07'59.4", E 101°40'08.1" | Urban |  |
| Jalan Genting | Federal Territory of Kuala Lumpur | N 3°12'22.7", E 101°43'31.2" | Urban |  |
| Putrajaya | Federal Territory of Putrajaya | N 2°58'09.0", E 101°41'27.4" | Wetland |  |
| Mersing | Johor | N 2°17'11.73", E 103°40'22.48" | Forest |  |
| Kluang | Johor | N 2°3'56.15", E 103°31'34.03" | Forest |  |
| Kota Tinggi | Johor | N 1°42'30.21", E 103°49'25.09" | Forest |  |
| Kudat | Sabah | N 6°46'06.1", E 116°47'10.9" | Village |  |
| Tawau | Sabah | N 4°35'51.9", E 117°35'05.8" | Forest |  |

**Additional file 1: Table S3.** *Anopheles* samples with *Plasmodium* infections.

|  | **Number of samples** | **Number of samples: stage/type of infection** |
| --- | --- | --- |
| *An. introlatus* | 28 | 17: *Plasmodium* DNA-positive whole mosquito  4: oocysts / *Plasmodium* DNA-positive abdomen  7: sporozoites / *Plasmodium* DNA-positive head/thorax |
| *An. latens* | 2 | 1: oocysts  1: *Plasmodium* DNA-positive abdomen |
| *An. balabacensis* | 2 | 1: sporozoites  1: oocysts |
| *An. umbrosus* | 2 | 2: oocysts |
| **Total** | 34 | 17: whole mosquitoes  9: oocysts / *Plasmodium* DNA-positive abdomen  8: sporozoites / *Plasmodium* DNA-positive head/thorax |
