# Supplementary figures and images for: Natural Wolbachia infection in field-collected Anopheles and other mosquito species from Malaysia
Source: Parasit Vectors. 2020 Aug 12;13:414. doi: 10.1186/s13071-020-04277-x (PMC7425011; doi:10.1186/s13071-020-04277-x)

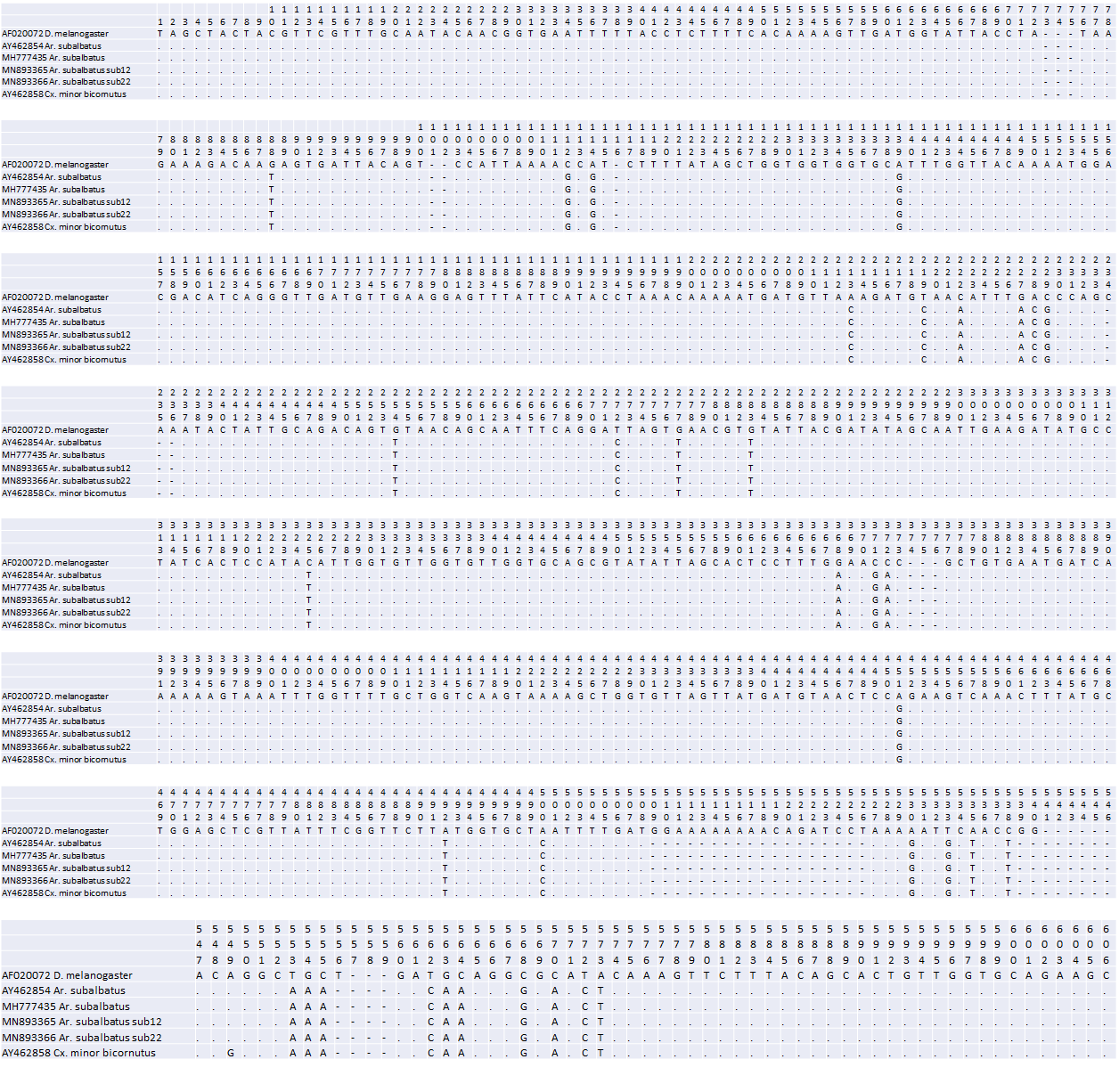

Supplement: Supplementary file 2 — Additional file 2: Figure S1. Alignment and variable sites of Wolbachia wsp sequences (Supergroup A) with nucleotide positions. [file 13071_2020_4277_MOESM2_ESM.tif]

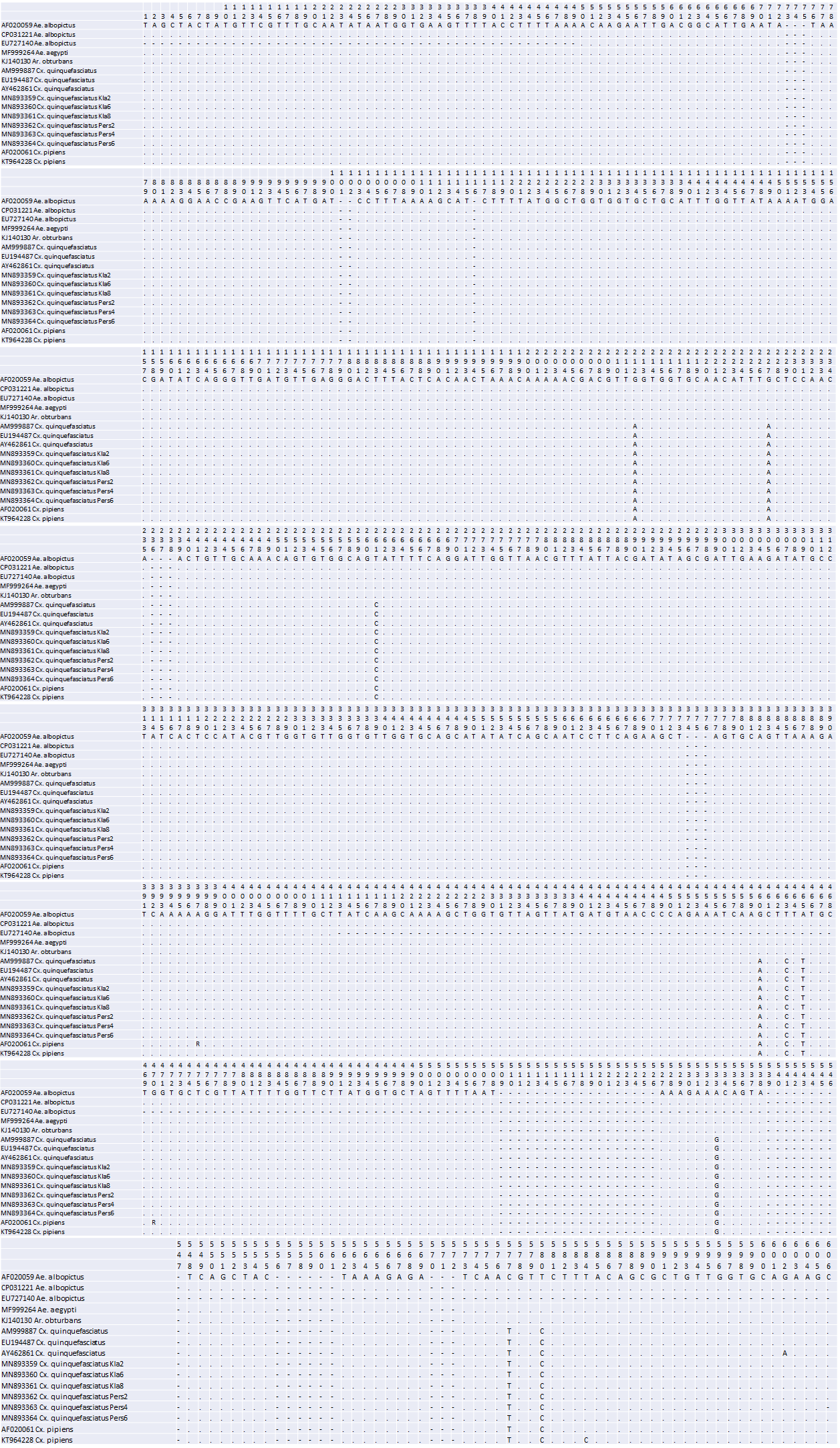

Supplement: Supplementary file 3 — Additional file 3: Figure S2. Alignment and variable sites of Wolbachia wsp sequences (Supergroup B) with nucleotide positions. [file 13071_2020_4277_MOESM3_ESM.tif]

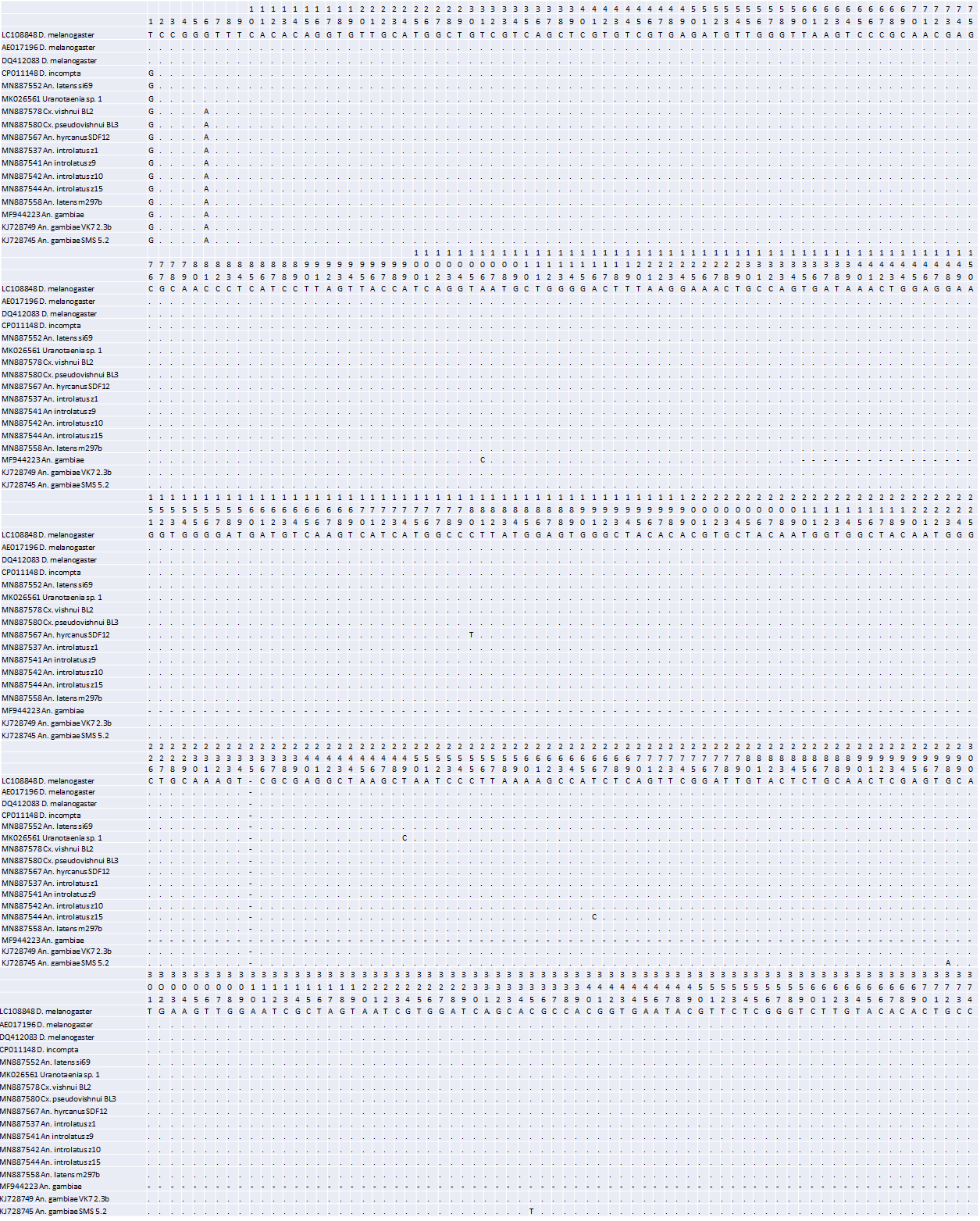

Supplement: Supplementary file 4 — Additional file 4: Figure S3. Alignment and variable sites of Wolbachia 16S rRNA (Supergroup A) sequences with nucleotide positions. [file 13071_2020_4277_MOESM4_ESM.tif]

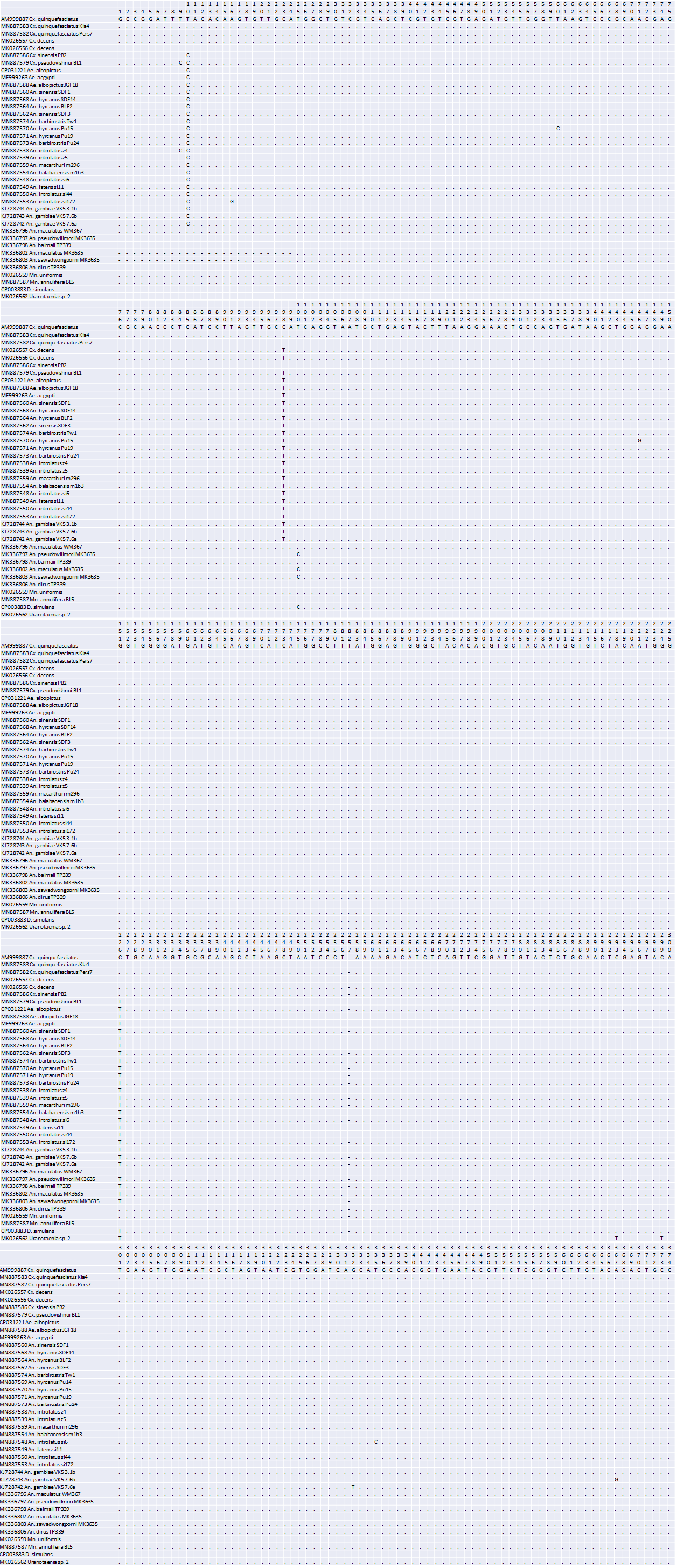

Supplement: Supplementary file 5 — Additional file 5: Figure S4. Alignment and variable sites of Wolbachia 16S rRNA (Supergroup B) sequences with nucleotide positions. [file 13071_2020_4277_MOESM5_ESM.tif]
